# Application of Nanoparticles in the Diagnosis and Treatment of Colorectal Cancer

**DOI:** 10.2174/0118715206323900240807110122

**Published:** 2024-08-08

**Authors:** Qiuyu Song, Yifeng Zheng, Guoqiang Zhong, Shanping Wang, Chengcheng He, Mingsong Li

**Affiliations:** 1 Guangdong Provincial Key Laboratory of Major Obstetric Diseases, Guangdong Provincial Clinical Research Center for Obstetrics and Gynecology, Department of Gastroenterology, The Third Affiliated Hospital of Guangzhou Medical University, Guangzhou, China

**Keywords:** Colorectal cancer, nanoparticles, nanomedicine, application, diagnosis, treatment

## Abstract

Colorectal cancer is a common malignant tumor with high morbidity and mortality rates, imposing a huge burden on both patients and the healthcare system. Traditional treatments such as surgery, chemotherapy and radiotherapy have limitations, so finding more effective diagnostic and therapeutic tools is critical to improving the survival and quality of life of colorectal cancer patients. While current tumor targeting research mainly focuses on exploring the function and mechanism of molecular targets and screening for excellent drug targets, it is crucial to test the efficacy and mechanism of tumor cell therapy that targets these molecular targets. Selecting the appropriate drug carrier is a key step in effectively targeting tumor cells. In recent years, nanoparticles have gained significant interest as gene carriers in the field of colorectal cancer diagnosis and treatment due to their low toxicity and high protective properties. Nanoparticles, synthesized from natural or polymeric materials, are NM-sized particles that offer advantages such as low toxicity, slow release, and protection of target genes during delivery. By modifying nanoparticles, they can be targeted towards specific cells for efficient and safe targeting of tumor cells. Numerous studies have demonstrated the safety, efficiency, and specificity of nanoparticles in targeting tumor cells, making them a promising gene carrier for experimental and clinical studies. This paper aims to review the current application of nanoparticles in colorectal cancer diagnosis and treatment to provide insights for targeted therapy for colorectal cancer while also highlighting future prospects for nanoparticle development.

## 1 INTRODUCTION

Colorectal cancer (CRC), a highly prevalent and fatal malignant tumor of the digestive system worldwide [1], ranks as the third most common and second deadliest cancer according to the World Health Organization (WHO) [2]. Lifestyle changes can significantly impact the prevention of colorectal cancer, with certain environmental factors playing crucial roles. Engaging in regular physical activity, consuming a diet rich in fiber, and taking calcium supplements serve as protective measures [3, 4]. Conversely, smoking, excessive alcohol intake, imbalances in gut bacteria (dysbiosis), and obesity are risk factors that increase the likelihood of developing colorectal cancer [3, 4].

CRC typically develops gradually from polyps, with an evolutionary process that can span 5-15 years, providing a feasible time window for early intervention and screening [5]. Early diagnosis of CRC is crucial for improving treatment outcomes and patient survival. However, early-stage CRC lacks typical symptoms or may even be asymptomatic, resulting in a low rate of early diagnosis. To improve the prevalence and coverage of early screening, there is an urgent need to find a new, sensitive, faster, non-invasive, and cost-effective method for detecting CRC. Treatment decision making and implementation are closely related to the prognosis and survival of patients diagnosed with CRC. The treatment methods of CRC are highlighted in Fig. (**1**). While the treatments have played a positive role in controlling disease progression and improving patient survival rates, their inherent limitations pose challenges in the management of CRC. Therefore, the field of CRC treatment urgently requires more personalized, effective and safe therapeutic options to overcome the shortcomings associated with existing treatments while enhancing patient quality of life, survival rates, and treatment response rates. With advancements in innovative medical research, particularly in nanotechnology and molecular biology, new possibilities have emerged for diagnosing and treating CRC.

Nanomaterials, a key element of nanotechnology, have made unprecedented breakthroughs in traditional medical treatments in recent years. As an emerging cutting-edge science and technology, nanotechnology has shown great potential, particularly in precision medicine and personalized treatment. Nanoparticles, as a core part of nanotechnology innovation, have emerged in the fields of medical diagnosis and therapy with promising applications. Innovative research on nanoparticles can contribute to the development of more precise and effective therapeutic strategies while promoting the dynamic combination of new materials, technologies, and medical innovations. A large number of studies have focused on innovative nanoparticle strategies for diagnosing and treating CRC. This review provides a brief summary of these studies and explores the properties, mechanisms, advantages, and challenges faced by nanoparticles with the aim of demonstrating their revolutionary role in managing CRC. Specifically, nanoparticles can improve diagnostic accuracy by enabling early detection while optimizing treatment regimens and reducing treatment-related toxicity.

## 2 NANOPARTICLES

### 2.1 Definition and Types of Nanoparticles

Nanoparticles, defined as tiny particles at the nanoscale with diameters ranging from 1 to 100 nm, are undoubtedly one of the current hotspots in medical materials research due to their unique physical, chemical, and biological properties, such as small-size effect, surface effect, and quantum size effect [5, 6]. Nanoparticles, with their diverse shapes, sizes, and structures, can be modified or encapsulated to improve biocompatibility and targeting capabilities [7]. This diversity in design, preparation, and application showcases their versatility [7]. The scalability and functional advantages of nanoparticles have demonstrated significant potential in precisely identifying diseases, advancing diagnostic methods, and boosting the efficiency of drug delivery [7]. This potential makes them a valuable tool in the development of more effective and targeted medical treatments.

Depending on the constituent materials, nanoparticles can be categorized into different types, such as metal nanoparticles, metal oxide nanoparticles, polymer nanoparticles, carbon-based nanomaterials, quantum dots and liposomes, and so on [8-15]. Metallic nanoparticles such as gold nanoparticles (AuNPs) and silver nanoparticles (AgNPs) are widely used in cancer therapy due to their excellent biocompatibility and unique optical properties. With the advantages of improved pharmacokinetics, flexible control of drug release, and ease of functionalization, metal nanoparticles show great potential in tumor immunotherapy [16]. Metal oxide nanoparticles such as titanium dioxide and cerium oxide can improve the hypoxic microenvironment of tumors, which can be applied to photodynamic therapy (PDT), and enhance the anti-tumor efficacy by modifying the nanoparticles to improve their targeting in cancer cells, inhibit tumor growth and metastasis [16]. Polymeric nanoparticles such as Poly lactic-co-glycolic acid and chitosan nanoparticles are applied in drug delivery systems due to their degradability and biocompatibility to achieve precise drug delivery and release by modifying antibody-targeting ligands [17]. Carbon-based nanomaterials such as carbon nanotubes and graphene are applied in photothermal therapy (PTT) of tumors and drug delivery due to their excellent electrical conductivity and mechanical properties [18]. Quantum dots are mainly used for highly sensitive imaging and tumor marker detection due to their unique fluorescent properties [19-21]. Liposomes are widely used for drug delivery and targeted therapies due to their efficient drug delivery capabilities and biocompatibility, which can significantly improve drug accumulation at the tumor site and reduce side effects [22, 23]. These different types of nanoparticles have a wide range of applications in cancer diagnosis and therapy due to their respective advantageous properties.

### 2.2 Targeting Strategies and Potential Applications of Nanoparticles

Nanoparticles, as an emerging drug delivery system, have a certain degree of customizability, and precise targeting of colorectal cancer cells can be achieved by improving the personalized design of nanoparticles. A variety of strategies and mechanisms for targeting CRC cells with nanoparticles are available: (1). surface modification and functionalization; (2). receptor-mediated endocytosis; (3). tumor microenvironment-responsive nanoparticles; (4). physical targeting strategies. The surface modification and functionalization strategy mainly involves the modification of specific ligands, such as antibodies, peptides, or small molecule drugs, on the surface of nanoparticles. These ligands can specifically recognize and bind to receptors overexpressed on the surface of CRC cells. Specifically, nanoparticles were modified using antibodies to carcinoembryonic antigen (CEA) or epidermal growth factor receptor (EGFR) to achieve their specific recognition and targeting of CRC cells [24]. Modification of nanoparticles with specific peptide sequences, *e.g*., RGD peptide-modified nanoparticles, can effectively target colorectal cancer cells expressing integrin αvβ3 [25]. Nanoparticles modified with folic acid can target CRC cells expressing folate receptor [26, 27]. Receptor-mediated endocytosis refers to the ability of nanoparticles, modified with specific ligands on their surface, to bind to specific receptors on the surface of colorectal cancer cells and be endocytosed by the cells, realizing efficient, targeted delivery of drugs. For example, transferrin-modified nanoparticles are able to enter CRC cells *via* TfR-mediated endocytosis [28]. Tumor microenvironment-responsive nanoparticles are designed to trigger drug release under specific conditions in the tumor microenvironment of colorectal cancer, achieving precision therapy [29]. This includes designing pH-responsive nanoparticles for the acidic environment or redox-responsive nanoparticles for the highly reducing environment in the tumor microenvironment. Physical targeting strategies, such as magnetic targeting, involve directing magnetic nanoparticles to accumulate at the tumor site under the influence of an external magnetic field, increasing the local concentration of the drug in the tumor and enhancing the therapeutic effect while reducing side effects [30-32]. Research on nanoparticles focuses not only on their synthesis and characterization but also on their behavior, efficiency, and possible health and environmental impacts in practical applications. The potential applications of nanoparticles in medicine are still in the developmental stage, and they have unique advantages in both diagnosis and treatment of diseases: (1) biomarker detection: early and non-invasive diagnosis of diseases through highly sensitive and specific detection techniques; (2) imaging: improving the resolution of imaging techniques and the sensitivity to disease markers; (3) drug delivery: the ability to efficiently carry and deliver drugs to specific body locations; (4) tissue engineering: supporting cell growth for repairing or replacing damaged tissues. In medical oncology, the advantages of nanoparticles in targeting and controlled release [33], as well as the integration of diagnosis and treatment, can better diagnose and treat tumors, meet the requirements of improving the efficiency of treatment under the premise of reducing the damage of treatment to normal healthy tissues, and optimize the strategy of cancer inhibition treatment. Accordingly, as a new medical material, the biocompatibility, long-term biosafety, and potential toxicity of nanoparticles are of concern, and in-depth research and optimization of the technology are still needed to realize the advantages of nanoparticles in cancer inhibition as soon as possible [34].

## 3 APPLICATION OF NANOPARTICLES IN THE DIAGNOSIS **OF CRC**

As medical technology continues to advance, nanotechnology has shown unprecedented potential in the field of cancer diagnosis, especially in the early detection and accurate diagnosis of CRC. Currently, traditional methods for screening, detecting and diagnosing CRC include blood tests (*e.g*., the CEA test), Digital rectal examination, Computed tomography (CT), magnetic resonance imaging (MRI), colonoscopy, *etc*. [35]. Despite their widespread use in clinical practice, these methods still have limitations, such as the insufficient sensitivity and specificity of traditional blood marker tests and the restricted capacity to detect early-stage tumors. Therefore, new technological innovations are urgently needed to overcome these challenges, in which the application of nanotechnology shows great potential, especially in improving the accuracy of early diagnosis.

Nanoparticles can be applied to the development of highly sensitive and specific biomarker detection platforms, providing higher sensitivity, specificity, and the simultaneous detection of multiple markers. This opens up limitless possibilities for early non-invasive detection of CRC [36]. In addition, nanoparticles can also target CRC cells using CRC-specific surface markers. It can be used in combination with imaging agents to prepare novel contrast agents, such as MRI contrast agents or fluorescent markers. What draws much attention to nanoparticles is their capability of improving the sensitivity and resolution of imaging techniques, enabling clear imaging, precise localization, and invasive range assessment of CRC. This not only assists clinicians in diagnosis, but also facilitates disease staging, treatment decision-making, and evaluation of treatment effects [37].

### 3.1 Application of Nanoparticles in CRC Biomarkers Detecting

Nanoparticle technology harbors tremendous potential in CRC biomarkers detection, notably for early diagnosis. Current screening and diagnostic practices, including colonoscopy, although recognized as the gold standard in CRC diagnosis, encounter limitations due to their high cost, complexity, and invasiveness, impeding their broad implementation in screening programs, particularly in resource-limited healthcare environments. Nanoparticle technology stands out for its exceptional sensitivity, specificity, cost-effectiveness, and non-invasive properties, presenting innovative solutions to these challenges. This technology is instrumental in developing advanced biomarker assays, facilitating early and accurate detection of CRC. Thus, focused research on nanoparticles' application in CRC biomarkers detection not only propels the advancement of early diagnostic methods but also fosters public adherence to comprehensive screening programs, significantly improving the management and prognosis for patients with CRC.

Tumor cell-derived exosomes, a subtype of exosomes, have a directional role in tumor development and degree of invasion. The development of novel tumor marker assays using CRC-derived exosomes as detection targets represents a cutting-edge research direction and application prospect, which is expected to bring about a revolutionary improvement in the diagnosis and management of CRC [38, 39].

A novel electrochemical biosensor developed by Wang *et al*. was aimed at detecting exosomes of CRC origin with a view to providing viable strategies and techniques for CRC detection [40]. It belongs to nanocomposites, prepared from p-sulfonamide [4] hydrated aromatic hydrocarbons (pSC4)-modified AuNPs and horseradish photo-oxidizing enzyme (HRP)-functionalized covalent organic frameworks, named HRPpSC4-AuNPs@COFs [40]. Experimental validation results showed that the biosensor has good biocompatibility and stability, and it is able to recognize and bind to the exosomes, with high detection sensitivity and selectivity, low detection baseline and wide detection range, which can successfully distinguish CRC patients from healthy people by clinical serum samples, and has potential in early diagnosis of CRC [40]. Sun *et al*. constructed a ratiometric fluorescent biosensor that achieved sensitive detection of the CRC-associated exosomal miR-92a-3p by utilizing self-assembled fluorescent AuNPs and duplex-specific nuclease (DSN)-assisted signal amplification [41]. Their experimental results showed that the biosensor had a good linear response over the concentration range of miR-92a-3p from 0.1 to 10 pM, with a detection limit as low as 45 fM, providing excellent stability and sensitivity. In addition, DSN-assisted signal amplification significantly improved detection sensitivity and specificity. The sensor is capable of distinguishing between CRC patients and healthy individuals and has a wide range of applications in the early detection and monitoring of CRC. Surface-Enhanced Raman Scattering (SERS) Biosensor can make amplification of originally weak Raman signals, facilitating the sensitive and specific detection of low concentrations of biomolecules [42, 43]. This bio-detection technology holds great potential for practical applications. Combining SERS technology with nanoparticles can further enhance its signal amplification. The surface plasmon resonance effect of nanoparticles, such as gold or silver nanoparticles, greatly enhances the SERS signal, increasing the sensitivity and specificity of biomolecule detection [44]. This makes SERS technology more efficient and sensitive in detecting biomarkers at very low concentrations, which is crucial for early diagnosis of diseases such as CRC. Therefore, SERS biosensors incorporating nanotechnology offer a very promising approach for the early detection and diagnosis of CRC.

It has been found that miRNA-21 and miRNA-31 are involved in the migratory and invasive behaviors of CRC and can be used as potential biomarkers for noninvasive screening, surveillance, and prognostic determination of CRC patients [45-47]. A biosensor targeting both CRC-associated miRNAs for quantitative detection and analysis was simultaneously designed and developed with the aim of providing a new detection strategy for early diagnosis and monitoring of CRC [48]. The biosensor represents a novel type of SERS biosensor [48]. It is composed of gold 3D hierarchically assembled nanocage@Au nanoparticles (AuNC@AuNP) coupled with silver-coated Fe_3_O_4_ magnetic nanoparticles (AgMNPs) [48]. This configuration achieves dual enrichment and amplification of SERS signals. The mechanism of combining nanoparticles with SERS biosensors for the diagnosis of CRC is shown in Fig. (**2A**). Through rigorous experimental validation, it has proven to be reliable, stable, reproducible, sensitive, and specific [48]. Notably, it has been confirmed to successfully differentiate CRC patients from the normal population based on specific detection targets [48]. Moreover, it provides valuable information for disease staging, highlighting its significant potential in the diagnosis and staging of CRC. It was found that the expression of nucleoside diphosphate kinase A (NDKA) was significantly increased in the blood of CRC patients, and thus NDKA was considered a clinically valuable CRC biomarker [49]. Based on this, Zhang *et al*. designed an immune-like sandwich multiple hotspots SERS biosensor for the detection of NDKA in serum [50]. This SERS biosensor employed an array of gold nanoparticles and specifically captured NDKA by imprinted polymer, NDKA antibody, and finally NDKA was detected by Raman signal. The biosensor was verified to have high sensitivity and specificity, which provided a new method and idea for the early diagnosis of CRC.

### 3.2 Application of Nanoparticles in CRC Imaging

In addition to showing its potential in the early detection of CRC, nanoparticle technology plays an important role in medical imaging. The introduction and use of nanotechnology contrast agents are transforming cancer imaging methods. They have improved imaging contrast, thereby significantly enhancing the performance of optical imaging techniques such as CT, MRI, and positron emission tomography (PET) [51]. More importantly, by binding to ligands, antibodies, or peptides with high affinity for tumor-specific markers, nanocontrast agents can specifically target tumor cells, thus providing greater clarity and accuracy of detection [51].

#### 3.2.1 Application of Nanoparticles in CT Imaging of CRC

There has been substantial advancement in the use of nanoparticles in CT imaging for CRC. The ability of nanoparticles to increase the sensitivity and accuracy of CT imaging has been amply demonstrated by numerous research advancements, highlighting the potential use of nanoparticles in the identification, accurate localization, and pathological staging of CRC.

An investigation was conducted on GdF3 nanoparticles enclosed in transferrin known as GdF3@Tf NPs [52]. For CRC imaging applications, the nanoparticles show good biocompatibility, effective multimodal imaging capabilities, and effective CT-enhancing effects [52]. They are very useful in preclinical imaging because of their precise targeting of transferrin receptors, especially in SW480 CRC models that overexpress transferrin receptors [52]. Because GdF3@Tf NPs target transferrin receptors, they can reduce toxicity and off-target effects while maintaining good safety and biocompatibility [52]. They are a flexible multimodal imaging contrast agents that have a clinical application promise for enhancing the precision of tumor staging and cancer detection [52]. However, the journey from preclinical success to clinical application necessitates rigorous pharmacokinetic and toxicological evaluations to fully understand the long-term safety and metabolic implications of these nanoparticles. As research progresses, it is anticipated that such innovative nanotechnologies will become integral components of personalized medicine, offering nuanced insights into tumor biology and facilitating the development of tailored therapeutic strategies.

#### 3.2.2 Application of Nanoparticles in MRI Imaging of CRC

MRI is a highly suitable and non-invasive imaging technique for soft tissue imaging, and its high-contrast imaging advantage helps to detect CRC and assess the staging, which has a crucial impact on the development of later therapeutic strategies [53, 54]. Integrating the advantages of nanoparticles with MRI imaging to develop contrast agents for imaging diagnosis of CRC is effective and feasible.

The fusion of antibodies specifically targeting vascular endothelial growth factor (VEGF) with nanotechnology for precise localization and imaging and treatment of tumors represents a highly selective targeting innovative technology strategy. The studies on VEGF have shown that in addition to its ability to affect tumor angiogenesis, VEGF can also modulate tumor-induced immunosuppression, making it a promising candidate target in the field of oncology therapy [55, 56]. This strategy enhances imaging precision and therapeutic efficacy through targeted attacks on VEGF while mitigating negative effects on healthy tissue. A study demonstrates the potential of anti-VEGF antibody-conjugated dextran-coated Fe_3_O_4_ NPs for CRC imaging [57]. The study successfully achieved actively targeted accumulation of CRC tissues in a CRC model by combining nanoparticles with VEGF antibodies, and significant imaging contrast effects were observed by MRI, demonstrating the great potential of anti-VEGF-NPs as molecularly-targeted tumor developers for MRI [57]. This result not only demonstrates the potential of combining nanotechnology and targeting factors in improving the diagnostic accuracy of cancer imaging but also provides new ideas for early cancer detection and treatment strategies [57]. The mechanism of combining nanoparticles with imaging technology for imaging diagnosis of CRC is shown in Fig. (**2B**).

Lectin-Conjugated Fe_2_O_3_@Au Core@Shell Nanoparticles have been developed to provide a viable option for dual-modality MR and CT imaging of CRC [58]. These nanoparticles were characterized by good biocompatibility and stability, as well as slight cytotoxicity [58]. In terms of imaging performance, these nanoparticles had good specific targeting ability in mice and were able to target and efficiently bind to SW620 CRC cells [58]. In addition, they exhibited significant MR and CT dual-mode imaging effects in both *in vivo* and *in vitro* studies, which is expected to become an MR/CT imaging of CRC effective targeting contrast agent applied in the early diagnosis of CRC [58].

Early monitoring of CRC liver metastases is beneficial for improving patient prognosis. The development of c-Met-targeting peptide-functionalized perfluoro-15-crown-5-ether nanoparticles (AH111972-PFCE NPs) provides a promising nanoradiographic agent for early monitoring of CRC liver metastases in imaging techniques. The nanoparticles showed high specificity for liver metastasis of CRC in *in vivo* and *in vitro* studies, strong c-Met targeting ability, and the ability to accurately detect tiny CRC liver metastases. In addition, the ultra-long tumor retention time combined with its strong c-Met targeting ability allowed the nanoparticles to highly selectively accumulate at the site of hepatic metastases and maintain a certain duration (at least 7 days) to monitor the efficacy of the therapy [59].

TRAIL-induced apoptosis is target-selective for many cancer cell types, and it has been recognized as a promising anticancer therapeutic strategy [60]. The development of a novel specific dual-targeted magnetic iron oxide nanoprobe has shown great potential in improving the sensitivity of MRI for CRC [61]. The nanoprobe consisted of magnetic iron oxide nanoparticles that underwent polyethylene glycolization as well as coupling with a bispecific fusion protein arginine-glycine-aspartate-tumor necrosis factor-related apoptosis-inducing ligand (RGD-TRAIL), which has excellent biocompatibility [61]. Polyethylene glycolization notably enhances the *in vivo* stability and biocompatibility of nanoprobes, enabling them to evade the body’s immune recognition and clearance, thus extending their circulation time in the bloodstream [61]. Additionally, attaching RGD-TRAIL to the nanoparticles allows the nanoprobe to specifically target the endothelium of tumor neovascularization and the tumor cell surface death receptors DR4 and DR5 [61]. This targeted approach significantly improves the sensitivity of MRI for COLO-205 CRC. Furthermore, the selective induction of apoptosis by RGD-TRAIL offers new avenues for tumor therapy, presenting a targeted strategy to combat cancer cells effectively.

#### 3.2.3 Application of Nanoparticles in PET Imaging of CRC

PET, an advanced nuclear medicine imaging technique, plays an important role in the diagnosis, efficacy assessment, and recurrence monitoring of CRC [62]. Fluorodeoxyglucose (FDG), one of the most commonly used radiotracers in PET imaging, can be taken up by cells without participating in the metabolism of glucose, resulting in its accumulative effect. Tumor cells with higher glucose uptake and metabolism rates exhibit increased FDG uptake and accumulation, leading to more pronounced high signals on PET scans [63-66]. The combination of nanoparticles and radioactive tracers such as FDG provides a new PET imaging method. These nanoparticles are designed to target specific tumor markers, thereby improving the specificity and accuracy of PET imaging diagnosis. This synergistic effect not only improves the sensitivity and specificity of imaging but also helps to gain a more comprehensive understanding of the biological behavior of tumors. Therefore, the fusion of PET technology and nanoparticles is a promising frontier in the continuous development of CRC imaging, with the potential to enhance early detection, treatment monitoring, and recurrence assessment of patients.

A dendritic macromolecular nanomaterial was designed and developed for PET imaging of CRC [67]. The permeation and retention effects of the nanomaterial as well as the dendritic multivalent and EPR-mediated tumor targeting achieved an effective accumulation of contrast agent at the tumor site, which significantly improved the sensitivity and specificity of PET imaging compared with the conventional contrast agent FDG [67]. The nanomaterial also provided a feasible solution for the PET imaging detection of tumors with a low need for glucose uptake [67]. A study by Boping Jing *et al*. demonstrated the development and application of a nanoprobe based on adipose-derived stem cell-derived extracellular vesicles for tumor visualization in an animal CRC model [68]. This innovative nanoprobe can be applied to PET/CT and near-infrared fluorescence imaging of tumors, achieving highly efficient tumor cell-specific targeting and clear imaging of CRC [68]. It can play a critical role in diagnosis of CRC imaging, preoperative evaluation, surgical pre-decision making, and intra-operative decision-making guidance, and is expected to improve surgical efficacy as well as patient prognosis and survival [68]. This study highlights the potential of extracellular vesicles as biocompatible nanocarriers in multimodal imaging techniques and provides a new strategy for accurate diagnosis and surgical guidance of CRC [68].

In addition, fluorescence imaging in the second near-infrared (NIR-II) window has made rapid progress in recent years. It has superior characteristics over traditional NIR-I imaging, including stronger tissue penetration depth, higher spatial and temporal resolution, *etc*., which enables clear visual imaging of tumor tissues, and these advantageous characteristics help the operator to more accurately differentiate the demarcation between normal healthy tissues and tumor tissues [69-72]. The combination of NIR-II and PET can integrate the advantages of both sides and improve the value of imaging technology in tumor diagnosis and treatment. Taking this as an entry point, Zhang *et al*. developed and prepared a hybrid NIR-II/PET bimodal nanoprobe with high fluorescence brightness, which has good performance in terms of stability, biocompatibility, loading efficiency, and flexibility and variability [73]. More importantly, the nanoprobe significantly enhanced the NIR-II/PET imaging contrast effect in the imaging of A431 tumor in mice, achieving clear visualization of the tumor site, confirming the high sensitivity and strong tissue penetration depth of the nanoprobe, suggesting that NIR-II/PET dual-modality imaging in high-quality imaging diagnostics of tumors with image-guided surgical precision optimization strategies showing good application prospects [73].

#### 3.2.4 Application of Nanoparticles in Other Imaging Techniques for CRC

Ultrasonography is an affordable, simple, rapid, safe and radiation-free adjunctive imaging diagnostic technique with unique value in the diagnosis and treatment of tumors [74]. The application of ultrasonography in the field of CRC mainly involves the evaluation of the infiltration depth and surrounding involved lymph nodes of CRC and ultrasound-guided needle aspiration biopsy by endoscopic ultrasound and transrectal ultrasound, which provide reference information for determining the accurate staging of CRC as well as formulating the corresponding treatment strategies [75].

Chen *et al*. developed an innovative nanoparticle system capable of transitioning from microbubbles to nanoparticles when triggered by ultrasound [76]. The transformed precursors, known as porphyrin/camptothecin-floxuridine triad microbubbles (PCF-MBs), serve as ultrasound/fluorescence dual-mode contrast agents for imaging [76]. This capability is anticipated to enhance the quality of tumor imaging, offering fresh insights for the early diagnosis of CRC. Furthermore, these converted nanoparticles show considerable potential for therapeutic applications, which will be elaborated in the subsequent therapeutic section.

In oncologic surgery, accurate identification and localization of the tumor and its boundaries are critical to ensure radical resection. Intraoperative tumor imaging technology enables surgeons to more accurately distinguish tumor tissue from normal tissue by providing real-time, high-contrast tumor visualization, enabling precise image-guided tumor resection [77]. The application of this precise tumor visualization technology not only helps in the accurate resection of tumors and reduces the risk of postoperative recurrence but also reduces the damage to healthy tissues, helps to preserve more functional tissues, and improves the quality of life of patients. Therefore, the development of efficient intraoperative imaging techniques is of great significance to increase the success rate of tumor surgery and improve the outcome and prognosis of patients.

CEA is a glycoprotein expressed in gastrointestinal tract tissues during embryonic development [78, 79]. CEA was initially identified as an antigen highly expressed in CRCs, but subsequent studies have found that it is also expressed in other types of cancers (*e.g*., breast, lung, pancreatic, and ovarian cancers) and therefore can be used as a tumor biomarker for cancer detection and monitoring [80, 81]. In the diagnosis of CRC, the sensitivity and specificity of CEA are not sufficient to support it as an important screening and diagnostic criterion, but for patients who have already been diagnosed with CRC, the level of CEA can provide referable information in monitoring the response to treatment, guiding therapeutic decisions, and predicting recurrence and prognosis [82-84].

A fluorescent silica nanoparticle conjugated to an antibody targeting CEA is able to bind specifically to CRC cells *via* the antibody and is intended to be applied to fluorescent imaging *in vivo* for visualization of the tumor site for real-time surgical guidance, thus increasing the precision and success of surgery and improving prognosis [85]. The results of the study showed that these nanoparticles successfully recognized and imaged CRC cells *in vivo* in CRC mice, demonstrating their great potential as an adjunct to cancer therapy [85]. This work not only provides a new strategy for tumor localization and surgical resection treatment of CRC but also opens up new avenues of using nanotechnology to target specific antibodies and other markers for specific imaging and treatment of other types of cancer.

In summary, the ideal imaging contrast agent for diagnostic medical imaging should have excellent properties, including high biocompatibility, low toxicity, good imaging effect, specific targeting ability, and appropriate half-life. In order to maximize the benefits of medical imaging in disease diagnosis, current research is aimed at improving the properties of contrast agents: improving their targeting, reducing or eliminating potential toxicity and side effects, and enhancing the strength and stability of imaging signals. The development of new material imaging contrast agents, including nanomaterials, is one of the hotspots of research, and various studies are constantly optimizing nanosystems to provide clearer imaging results and a wider range of applications. In this process, the development of developers based on novel materials, especially those related to nanotechnology, has become a hot research topic. These novel nano developers are being intensively studied and optimized to achieve more precise and clearer imaging results, expanding their potential in various medical applications.

## 4 TREATMENTS FOR CRC

### 4.1 Application of Radiotherapy in the Treatment of CRC

Radiotherapy uses high-energy radiation to directly target the tumor area, causing damage to the DNA of cancer cells. This ultimately leads to the eradication of cancer cells and the control of tumor growth and metastasis. Radiation therapy has irreplaceable advantages in improving the local control rate and prolonging the survival period [86-90]. However, radiotherapy for CRC still has certain therapeutic limitations, and further research and technological innovation are needed. The application of nanomedicine in exploring and developing novel radiosensitizers represents a cutting-edge direction aimed at improving the efficacy of radiation therapy. This is achieved by increasing the sensitivity of cancer cells to radiation while minimizing damage to normal tissues.

Darmon *et al*. designed a radiosensitizer for radiation therapy of colorectal cancer, hafnium oxide nanoparticles (NBTXR3) [91]. NBTXR3 is a functionalized hafnium oxide nanoparticle with a high electron density, which significantly increases energy deposition inside cancer cells. Compared to radiotherapy alone, NBTXR3, in combination with radiotherapy, was able to significantly reduce cancer cell survival and enhance the rate of apoptosis and necrosis in both early and advanced stages. In addition, this study demonstrated that NBTXR3 not only enhanced the direct killing effect of radiotherapy but also produced systemic anti-tumor effects by enhancing anti-tumor immune responses. Overall, NBTXR3, as an effective radiation enhancer, demonstrated significant anti-tumor effects and immune activation effects in preclinical studies.

Radiation resistance is one of the limitations of radiotherapy. Overcoming or reducing radioresistance is an important issue in current cancer research [92]. To address the important issue of radioresistance, Lee *et al*. argued that the development and application of radioresistance diagnostic probes can screen the population of radioresistant CRC in actual clinical diagnosis and treatment, personalize treatment decisions, and improve the efficiency of radiation therapy, which can help to maximize the therapeutic benefits [93]. They constructed radioresistant CRC tissues in a patient-derived tumor xenograft mouse model and identified low-density lipoprotein receptor-related protein-1 (LRP-1) as a CRC radioresistance marker protein [93]. Then, they designed and developed LRP-1-targeted 5-FU-containing human serum albumin nanoparticles with the aim of highly targeting to improve the radioresistant CRC neoadjuvant radiotherapy effect [93]. In addition, the nanoparticles can highly target and improve the effectiveness of neoadjuvant radiation therapy for radiation-resistant CRC and enhance the ability to monitor CRC treatment response through specific diagnostic imaging techniques [93]. In summary, this strategy helps to achieve real-time evaluation of early diagnosis and treatment effectiveness, providing more accurate and personalized treatment plans for CRC patients.

### 4.2 Application of Chemotherapy in the Treatment of CRC

While radiation therapy plays an important role in targeting and controlling localized areas of CRC, a combination of chemotherapy is often needed to attack the cancer cells and reduce the risk of systemic recurrence in order to treat it fully. Chemotherapy is a treatment to suppress cancer through the systemic or local use of chemicals. Chemotherapy mainly kills, controls, or slows down the growth of cancer cells by interfering with the growth and division process of the cancer cells in order to effectively control the growth of the tumor or kill the tumor. Chemotherapy, one of the important therapeutic strategies for CRC, is usually used in combination with other treatments, such as surgery and radiotherapy, to improve the cancer suppression effect [94]. However, the challenge of chemotherapy resistance poses a significant problem in the treatment of CRC [95]. An in-depth exploration of the mechanisms of chemoresistance is an important prerequisite for improving the efficacy of chemotherapy and overcoming its limitations. Many studies have made important contributions to revealing the molecular mechanisms of chemoresistance [95]. Although extensive research has been conducted on these resistance mechanisms, there is still ongoing research and development of new strategies specifically targeting these mechanisms to overcome resistance. This includes the development of new drugs [96] and the use of drug-combination therapies [97]. Targeted therapies and nanomedicinal drug-delivery systems for specific cancer cell markers [98] represent crucial research directions in the field of cancer therapy.

He *et al*. found that BRD4 expression was upregulated in CRC treated with doxorubicin [99]. BRD4 is a member of the bromodomain and extra-terminal family of proteins [100]. It plays an important role in a variety of biological processes, including cell cycle regulation, cell proliferation, and inflammatory responses [101-103]. In addition, the aberrant expression or activity of BRD4 in certain cancer types has been associated with cancer development [104, 105]. They hypothesized that this was likely a potential resistance mechanism for tumors treated with doxorubicin [99]. As a starting point, they constructed a heterobifunctional BRD4 molecular degradation drug, ARV-825, with doxorubicin in CRGD-modified nanoparticles [99]. Taking this as an entry point, ARV-825, a heterobifunctional BRD4 molecular degrading drug, was loaded with doxorubicin in CRGD-modified nanoparticles to construct tumor-targeting nanoparticles, ARV-DOX/cRGD-P, which were used in the treatment of CRC99. Subsequent experiments affirmed the safety and stability of this nanosystem. They also verified that the ARV-DOX/cRGD-P complex has the ability to inhibit tumor proliferation and angiogenesis and exhibit good anti-tumor efficacy in doxorubicin chemotherapy regimen for CRC [99]. These findings significantly improved treatment sensitivity and demonstrated a promising new targeting chemoresistance mechanism of anti-tumor nanomedicine. Lang *et al*. constructed a nanoparticle (SCXN) consisting of amphiphilic derivatives synthesized from xylan-stearic acid and capecitabine-stearic acid [106]. These nanoparticles were effective in prolonging the half-life of capecitabine in the blood and increasing the accumulation of the drug in tumors. SCXN not only acted as a drug carrier but also functioned as a prebiotic, promoting the proliferation of beneficial bacteria such as Bifidobacterium and Roseobacter and increasing the production of short-chain fatty acids. This all helped to inhibit tumor development and enhanced the anti-tumor immune response. Compared to conventional capecitabine therapy, the use of SCXN improved the anti-tumor effect of the drug and reduced dose-related toxicity. This suggests that nanoparticle systems have important applications in improving drug efficacy and safety.

Nanoparticles offer a promising approach for targeted drug delivery to treat tumors due to their good drug targeting and bioavailability. Additionally, they can enhance the penetration and retention effects of drugs, control the timing of drug release, and reduce multidrug resistance [107, 108]. Zhao *et al*. [109] developed gold nanoparticles to enhance the efficacy of cisplatin chemotherapy in treating CRC. These nanoparticles were found to reduce the expression of pro-fibrotic signals, such as CTGF, TGF-β1, and VEGF, through the Akt signaling pathway. Additionally, they decreased the density of cancer-associated fibroblasts and tumor stromal collagen I. As a result, there was a significant reduction in the solid stresses exerted by tumor mesenchymal mechanisms on vasculature. Ultimately, this improved perfusion and hypoxia levels within the tumor microenvironment and significantly enhanced cisplatin delivery efficiency. Consequently, it amplified the effectiveness of nanoparticle-assisted chemotherapy strategies for treating CRC [109].

Targeting tumor endothelial cells for cancer inhibition therapeutic strategies holds promise due to their more stable expression of surface receptors compared to tumor cells, greater accessibility to chemotherapeutic agents in the bloodstream, and reduced susceptibility to drug resistance [110-112]. E-selectin is overexpressed on the tumor vascular endothelial cells, and its high expression has been unequivocally associated with tumor vascular growth and metastasis [113, 114]. In CRC, the binding of E-selectin to ligands has been found to promote invasion and metastasis of CRC cells [115]. Hao *et al*. developed a novel nanoparticle targeting tumor vasculature using E-selectin as a target [110]. They first prepared an amphiphilic polyethylene glycolated peptide-drug coupling by coupling an E-selectin-binding peptide, polyethylene glycol (PEG), with the chemotherapeutic drug SN38, and then further prepared the coupling into new self-assembled nanoparticles [110]. The nanoparticles were able to target the tumor vascular endothelium and efficiently release the chemotherapeutic drug SN38 to the tumor region in the tumor microenvironment with high glutathione concentration, which significantly inhibited tumor growth, suppressed distant lung metastasis, and prolonged survival in a mouse CRC model [110]. These findings highlight the promising antitumor activity of the nanoparticles, suggesting that they can be used as an adjuvant synergist for chemotherapy in combination with chemotherapeutic agents to improve drug delivery efficiency and provide an effective and feasible treatment strategy for CRC patients [110].

Nanoparticle therapy on tumor vascular endothelial cells shows remarkable potential to inhibit tumor growth and metastasis. It is also noteworthy that the regulation of the intestinal microenvironment also plays an important role in the treatment of CRC. Short-chain fatty acids produced by beneficial intestinal flora play an important role in regulating intestinal microecology, maintaining intestinal barrier homeostasis, and modulating intestinal immune responses [116]. Modulating the intestinal microbiota to promote the growth of oncogenic flora while inhibiting the survival of pro-oncogenic flora represents a potential therapeutic strategy for CRC. Clostridium nucleatum, a well-established pro-tumorigenic bacterium, is strongly associated with CRC development, distant liver metastasis, and resistance to chemotherapy [117-121]. Zheng *et al*. took advantage of the selectivity and specificity of phage for bacteria to develop irinotecan-loaded dextran nanoparticles covalently linked to azide-modified phage [122]. The nanoparticles, guided by phages and specifically targeted against C. nucleatum, were combined with irinotecan’s effective killing of CRC cells and dextran’s regulation of intestinal flora as well as promotion of short-chain fatty acid production [122]. This combination promoted the propagation of beneficial bacteria like Bacillus butyricus while destroying C. nucleatum, ultimately significantly improving chemotherapy efficacy in treating CRC [122]. This study provides a promising therapeutic strategy that can effectively improve the efficacy of chemotherapy for CRC by selectively modulating the gut microbiota, thereby highlighting the potential of microbiota-targeted therapies in oncology.

The quest for CRC treatment is not limited to the gut microbiology level. In fact, the use of nanotechnology in combination with ultrasound technology also shows great potential as an effective new strategy. Chen *et al*. combined nanotechnology and ultrasound technology to develop an innovative nanoparticle converted from microbubbles under ultrasound triggering mechanism [76]. Under the ultrasound triggering mechanism, their prepared PCF-MBs could be converted into PCF-NPs. These converted nanoparticles enhanced the permeability of capillary walls and cell membranes through the ultrasound perforation effect, thus facilitating the precise accumulation of chemotherapeutic drugs and photosensitizers at high concentrations in the tumor [76]. *In vivo* experimental results also demonstrated a significant cancer inhibition efficacy (with a tumor inhibition rate of 90%) when the nanoparticles were combined with ultrasound and laser irradiation without any notable recurrence [76]. In addition, they found that the nanoparticles significantly inhibited the expression of adenosine triphosphate-binding cassette subfamily G member 2, a factor associated with drug resistance in tumors. This inhibition helped overcome multidrug resistance and enhanced the effectiveness of chemotherapy [76]. In addition to its previous potential in diagnostic imaging, the study by Chen *et al*. demonstrated a novel approach to convert therapeutic drug-rich microbubbles into nanoparticles through ultrasonic triggering. This not only enhances contrast and clarity for imaging but also improves tumor targeting of drugs, providing a new strategy for early diagnosis and treatment of CRC. The findings highlight the significant potential of nanotechnology in enhancing both imaging diagnostic efficiency and therapeutic efficacy [76].

### 4.3 Application of Phototherapy in the Treatment of CRC

Phototherapy mainly includes PTT and PDT, both of which have been proven to be highly effective in treating CRC [123]. Nanoparticle-based photosensitizers show great potential for optimizing the treatment strategies of phototherapy, as they offer enhanced precision, safety, and efficiency in targeting tumor [124]. The mechanism by which nanoparticles synergistically enhance PDT treatment for CRC is illustrated in Fig. (**3**).

#### 4.3.1 Application of PTT in the Treatment of CRC

PTT is a treatment that utilizes a light absorber that generates heat when irradiated with a specific wavelength of light, thereby selectively inducing apoptosis or necrosis of cancer cells. The absorption and scattering of the near-infrared laser used in photothermal therapy by normal tissues lead to a decrease in the thermal energy converted from light energy in the tumor target tissues, so the actual effective rate of cancer inhibition decreases accordingly. It is difficult to control the damage to normal tissues by increasing the light energy so that the efficacy of the therapy is obviously limited [125]. In PTT, NIR-absorbing materials can reduce the absorption of light energy in normal tissues to avoid the accumulation of heat energy in normal tissues while generating sufficient heat in the local tissues of tumors to treat cancer [126, 127].

Combining the advantages of nanoparticles with photothermal therapy is a key area of research. The goal is to synthesize and prepare nanoparticle polymers with superior photosensitivity and photothermal conversion efficiency [127]. These polymers are used for cancer imaging diagnostics and therapy. This research is an important part of employing nanomaterials as a popular technology to overcome tumor challenges. Previous studies have demonstrated the ability of nanoparticles synthesized on this basis to reduce tumor size and act as a cancer suppressor [128, 129]. McCabe-Lankford *et al*. also developed two NIR-absorbing novel polymeric nanoparticles on this basis, with PCPDTBSe used for photothermal therapy to enhance the therapeutic efficacy of the treatment and H-DAPPs used for fluorescence imaging using photothermal therapy as a combined platform [125]. Both nanoparticles were able to act by highly selectively targeting tumor tissue [125]. H-DAPPs are nanoparticle mixtures of non-fluorescent PCPDTBSe and fluorescent PFBTDBT10 assembled in a ratio of 1:19, which are able to fulfill the dual requirements of photothermal therapy and fluorescence imaging at the same time [130]. Both the final temperature received by the tumor tissue and the uniformity of the heat aggregation distribution are key factors affecting the cancer inhibition effect of photothermal therapy [128]. What can be ascertained after the evaluation of alginate tissue phantom and 3D tumor spheroid assay is that both polymer nanoparticles have good heating performance as well as superior ability to kill CRC cells in an *in vitro* model under the laser parameters (100 μg/ml nanoparticles, 2.2 W/cm^2^, 60 s, 800 nm) [125]. Due to one more advantageous feature of fluorescence visualization over PCPDTBSe, H-DAPPs are able to perform the dual tasks of photothermal therapy against tumors as well as tumor visualization and detection/diagnosis [125]. They are attractive star nanomaterials combining therapeutic and visual detection of CRC and even other types of tumors with a bright prospect in the future [125].

In addition, there are many other nanoparticles developed in many research institutes with good cancer inhibition performance in photothermal therapy for CRC. Xing *et al*. developed upconversion nanoparticles coated with polyaniline with upconversion luminescence and photothermal conversion properties (UCNPs-PANPs) for photothermal therapy of cancer [131]. These low-toxicity nanoparticles are biocompatible and capable of converting light energy into thermal energy, effectively ablating the cancer cells and providing imaging capability for therapeutic guidance, presenting a significant targeted cancer therapeutic potential [131]. Polydopamine-coated Au-Ag nanoparticles have shown low cytotoxicity and high biocompatibility in both *in vitro* and *in vivo* models [132]. They were found to be able to induce cell death *via* cystatinase-dependent and non-dependent apoptosis, mitochondrial damage, lysosomal membrane permeability, and autophagy and are suitable for use in the treatment of CRC cancers with PTT [132]. McCarthy *et al*. demonstrated the potential of semiconductor polymer nanoparticles for effective targeting and photothermal ablation of CRC cancer cells in a 3D organoid model using the tumor organoid technique, underscoring the value of their application in CRC treatment [133].

#### 4.3.2 Application of PDT in the Treatment of CRC

PDT is a method of treating cancer through the interaction of photosensitizers, specific wavelengths of light and oxygen molecules. When the photosensitizer accumulates in the tumor or diseased tissue and is irradiated by light of a specific wavelength, the photosensitizer reacts with oxygen and produces mono-linear oxygen or reactive oxygen species. These reactive oxygen molecules are capable of directly destroying the structure and function of tumor cells, ultimately leading to apoptosis or necrosis.

The integration of nanoparticles with PDT holds significant promise for advancing CRC treatment. This field has seen extensive research focused on enhancing and refining these methods. CEA is highly expressed in most colorectal cancers and is an ideal targeting marker. Khaled *et al*. synthesized a silica-based nanoparticle and functionalized the anti-CEA Affimer protein on its surface [134]. In *in vitro* experiments, the functionalized nanoparticles demonstrated significant CEA-specific fluorescence, which could avoid non-specific accumulation in normal tissues, thus reducing potential side effects. In PDT experiments, anti-CEA-Affimer functionalized nanoparticles were able to significantly induce cancer cell death and significantly promote tumor volume reduction. Overall, their study demonstrated that CEA-Affimer-based Foslip nanoparticles have significant advantages in imaging and PDT of colorectal cancer, offering new possibilities for personalized treatment of colorectal cancer. Folate receptor is highly overexpressed on the surface of CRC cells, and targeting CRC cells with folic acid can improve the selectivity of PDT to tumor tissues [135]. Nandi *et al*. [136] assembled and synthesized folic acid FA and ferric oxide Fe_2_O_3_ to prepare folic acid template water-soluble magnetic iron oxide NPs (FA7Fe_2_O_3_). The nanoparticles were characterized by low toxicity, high stability, and more intriguingly, good performance and performance in PDT targeting inhibition of human CRC cell line (HCT 116) as well as diagnostic CRC applications [136]. The mechanism of inhibition of CRC by photothermal therapy with FA7Fe_2_O_3_ nanoparticles is to induce the generation of ROS (mainly hydroxyl radicals), which directly damages the nuclear DNA of tumor cells and mediates the phosphorylation of p53 [136]. The nanoparticles are able to promote the expression of pro-apoptotic proteins, inhibit the expression of anti-apoptotic proteins and then initiate the mitochondria-dependent apoptotic pathway, which ultimately leads to the death of the tumor cells [136]. The mechanism by which these nanoparticles promote apoptosis of cancer cells in PDT is shown in Fig. (**4**). In addition, *in vitro* MRI tests have also confirmed that FA7Fe_2_O_3_ can simultaneously play a potentially important role as a T2 contrast agent in the diagnosis of CRC [136]. This nanosystem integrates diagnostic and therapeutic applications and shows great promise in the application of CRC diagnosis and treatment [136].

Bufotalin has been discovered to inhibit the proliferation of cancer cells and block tumor migration and invasion [137]. Additionally, it affects angiogenesis and the immune microenvironment, establishing it as an innovative and promising candidate for cancer therapy [137]. Meta-tetrahydroxyphenylchlorin (mTHPC), also known as Foscan, is a second-generation photosensitizer noted for its high effectiveness [138]. It possesses the ability to kill tumor cells and activate the immune system [138, 139]. Given its low toxicity and safety for normal tissues, mTHPC shows promise for the PDT of specific cancers, particularly in treating head and neck tumors [138, 139]. Previous studies by Yuan *et al*. have shown that toadoxin can play an adjuvant role in promoting cancer inhibition in PDT targeting CRC cells with mTHPC as a photosensitizer. Yuan *et al*. prepared IRGD-modified VEC-CSO/TPGS-RGD multifunctional targeted nanoparticles, which were loaded with BUs and mTHPC photosensitizer for experimental study in order to explore the mechanism behind it and further improve the curative effect of PDT [140]. IRGD, a tumor-homing peptide, enhances the transport of drugs and nanoparticles by binding to tumor vasculature [141, 142]. This binding facilitates the diffusion of drugs and agents into the tumor parenchyma, improving drug penetration and targeting of cancer cells and tumors [141, 142]. Consequently, it aids in increasing the efficacy of PDT and reducing tissue side effects. Experimental results confirm that VEC-CSO/TPGS-RGD multifunctional targeted NP is an efficient, safe, and nontoxic BU and mTHPC co-delivery nanomedicine system, which is able to improve the solubility of BU and mTHPC and increase the targeting co-delivery efficiency [140]. The nanoparticle-loaded BU targets SRC-3 in CRC cells, thereby inhibiting the formation and maintenance of the tumor hypoxic environment [140]. This action leads to reduced expression of downstream HIF-1αand VEGF [140]. As a result, apoptosis induced by PDT, with mTHPC as the photosensitizer, is enhanced, ultimately improving the antitumor efficacy of mTHPC-PDT in CRC [140]. This study not only provides a promising strategy to enhance the role of PDT in CRC treatment but also reveals the signaling pathway mechanism by which PDT synergized by toadstool and mTHPC inhibits CRC cells [140]. Ostroverkhov *et al*. developed bacteriochlorin-loaded magnetic nanoparticles (MNPs) capable of MRI-guided targeted delivery of Hydrophobic bacteriochlorin based photosensitizer to the tumor site, which enhances the solubility of the photosensitizer while preserving its photodynamic activity [143]. In studies using CT26 mouse colon cancer cells, both *in vitro* and *in vivo*, the time-dependent accumulation of these nanoparticles at the tumor site enabled efficient delivery of the photosensitizer [143]. This resulted in significant photo-induced toxicity, thus enhancing the therapeutic efficacy of PDTy for CRC [143].

The generation of ROS by PDT to exert cancer inhibition requires the critical involvement of oxygen. However, local hypoxia in tumor tissues due to rapid tumor growth and division is the norm in the microenvironment of most solid tumors. The consumption of oxygen by the PDT process will further exacerbate the degree of local hypoxia in tumors, which ultimately leads to a significant reduction in the therapeutic effect of PDTs [144-150]. In special circumstances, it even promotes the invasion and metastasis of the tumors, resulting in the PDT treatment limitations of tumors [144-150].

Perfluorocarbon@Porphyrin Nanoparticles developed by Liang *et al*. were designed to enhance the efficacy of PDT in the treatment of liver metastasis of colon cancer by improving the hypoxia of the tumor microenvironment as an entry point [151]. The innovative aspect of this nano-delivery system is its ability to dual-deliver both the photosensitizer and oxygen directly to the tumor site [151]. By replenishing oxygen, it enhances the efficacy of PDT, inhibiting tumor growth, reproduction, and metastasis [151]. The nanoparticles can also be used as a bimodal contrast agent for fluorescence and CT imaging for diagnostic imaging of CRC liver metastases, which can help to accurately guide PDT by enabling visualization of the tumor site and real-time monitoring of the treatment process [151]. The development of this nanosystem introduces a method for real-time monitoring and precise targeting of therapy, an innovation that represents a promising advancement in cancer treatment and provides a more effective and targeted approach to combat tumor growth and metastasis [151].

X-ray induced photodynamic therapy (X-PDT) is an innovative therapeutic approach aimed at overcoming the limited depth of light penetration, a major limitation encountered by conventional PDT in the treatment of deep tumors [152, 153]. X-PDT leverages the high penetrating power of X-rays to enable PDT to reach the deep tumor locations that are difficult to reach with conventional PDT [154, 155]. In a study by Gong *et al*., hollow polydopamine-conjugated platinum nanoparticles (Pt@HP) and AVPt@HP@M, loaded with apoptin and verteporfin, were developed, offering a novel therapeutic strategy for the radiotherapy of CRC [156]. This nanoplatform utilizes the peroxidase-like activity of platinum nanoparticles, which promotes the in situ generation of oxygen, improving the hypoxic environment within the tumor [156]. Additionally, it carries AP and the photosensitizer VP, enhancing both the apoptotic and photodynamic therapeutic effects [156]. The nanoplatform demonstrates significant radiosensitization effects in both *in vitro* and *in vivo* models, potentially enhancing CRC radiotherapy [156]. It employs multiple strategies, including alleviating hypoxia, promoting tumor apoptosis, and utilizing X-PDT based on the mechanism of purine metabolism [156]. In addition, the platform demonstrated good biocompatibility and safety as well as targeting ability, providing a new way to sensitize radiotherapy for malignant tumors such as CRC [156].

### 4.4 Application of Immunotherapy in the Treatment of CRC

In the treatment of CRC, the fusion of nanotechnology with immunotherapy represents a groundbreaking advancement. This approach not only enables precise targeting of cancer cells but also invigorates and amplifies the body's immune response. Tumor immunotherapy is an important therapeutic strategy to inhibit tumor development by activating the body's own immune system to complete the recognition and, killing and removal of cancer cells [157-159]. Immunotherapy has become an important tool in the treatment of CRC, especially for some patients with high microsatellite instability CRC expressing specific immune checkpoints such as programmed cell death-1 (PD-1) or programmed death ligand-1 (PD-L1). For these patients, immunotherapy can offer the possibility of long-term control or even cure [160, 161].

Wang *et al*. developed a silica body nanoparticle carrier, a mesoporous silica nanoparticle coated with a lipid bilayer, capable of delivering irinotecan (IRIN) and combined with radiation therapy for CRC treatment [162]. The combination therapy demonstrated superior antitumor efficacy compared to monotherapy. Intriguingly, it not only synergistically enhanced the therapeutic effects inherent to the combination of radiotherapy and chemotherapy but also significantly increased the sensitivity to immunotherapy. The synergistic therapeutic effects of radiotherapy induced DNA damage, facilitating the activation of the intracellular cGAS/STING pathway [162]. This resulted in increased expression of major histocompatibility complex class I and PD-L1 and a significant rise in the number of tumor-infiltrating CD4+ T cells, CD8+ T cells, and dendritic cells [162]. Consequently, there was an enhancement in tumor antigen presentation and activation of the immune microenvironment. These activating effects on the immune system significantly boosted the efficacy of anti-PD-1 immunotherapy for CRC. This novel therapeutic strategy underscores the potential of integrating nanotechnology with radiotherapy and chemotherapy. Leveraging the unique properties of nanoparticles, it aims to generate a targeted and enhanced response to CRC. Furthermore, this approach demonstrates a promising direction for future cancer treatments that more efficiently utilize the human immune system, thereby sensitizing tumors to immunotherapy [162].

Cancer cell death can be categorized into two types: immunogenic and non-immunogenic [163]. Immunogenic cell death (ICD) refers to a type of cell death that stimulates the immune system's response. This stimulation occurs through the release of signaling molecules known as “death-associated molecular patterns.” These molecules attract and activate dendritic cells and T cells within the immune system, leading to a specific immune response that inhibits and clears tumors, which is beneficial in preventing the development and spread of cancer [164-167]. The concept of ICD offers a new strategy for cancer treatment by enhancing the body's immune response to tumors through the induction of cancer cell death, thus improving the efficacy of immunotherapy. Building on this, Li *et al*. developed a nanoparticle (GCMNPs) that, when combined with iron therapy to induce ICD, can significantly boost immunotherapy for CRC [168]. This enhancement is achieved by blocking the PD-1/PD-L1 pathway, a critical component of immune checkpoint therapy, thereby inducing effective tumor-specific immunogenic cell death [168].

Dysregulation of intestinal flora has been recognized as an important factor influencing the occurrence and development of CRC. Maintaining a healthy state of intestinal flora is of significance in reducing the risk of CRC, and may serve as a potential strategy for the prevention and treatment of CRC [169, 170]. Lipopolysaccharide (LPS), one of the cell wall components of intestinal Gram-negative bacteria, has been shown to play a key role in promoting inflammatory responses and immune regulation [171]. In immunotherapy for CRC, LPS-induced inflammatory responses may promote immunosuppression in the tumor microenvironment, thus providing tumor cells with opportunities to evade immune surveillance [172]. Reducing LPS-induced inflammation could help reduce immunosuppression and enhance tumor sensitivity to immunotherapy. Song *et al*. identified high levels of LPS in CRC tissues and designed and developed a lipid-protein-protein-dNA nanoparticle system incorporating LPS-targeted fusion proteins [172]. In a CRC model, the nanoparticle system effectively enhanced the immunotherapeutic efficacy of the PD-L1 monoclonal antibody against CRC by targeting LPS to block its immunosuppressive effect [172]. Additionally, it inhibited liver metastasis of CRC [172]. This demonstrates a promising approach to targeting the gut microbiome as a therapeutic target for the focused treatment of CRC.

### 4.5 Application of Gene Therapy in the Treatment of CRC

Gene therapy is a novel therapy for disease intervention at the gene level, aiming to prevent or treat diseases by restoring the function of damaged genes, inhibiting the expression of disease-related genes, or introducing new genes. In recent years, the development of gene therapy has been rapid, and a variety of gene therapies have been formally approved for clinical use [173], in addition to a variety of gene therapies that are in the research and development or clinical trial stage. In the field of cancer, gene therapies approved for clinical application in head and neck cancer, nasopharyngeal cancer, and pancreatic cancer have existed [174], and gene therapy has great potential for therapeutic application in CRC, and related research is still in the stage of clinical development and trials. mRNAs, miRNAs, and siRNAs in RNA are potential candidates for gene therapy, and nanosystems combining nanoparticle-targeted delivery of drugs have been co-assembled. The nanosystems gene therapy platform co-assembled with the advantages of nanoparticles for targeted drug delivery opens up a new direction for gene therapy for CRC.

Aghamiri *et al*. [175] discussed in detail the different strategies and challenges of using nanoparticle delivery of siRNAs for the treatment of CRC. They emphasized that the synergistic effect of gene therapy and chemotherapy can overcome the limitations of multidrug-resistant chemotherapy and that targeting specific molecules, such as siRNAs, by nanoparticle systems is able to silence oncogenes and multidrug-resistance (MDR)-related genes in tumor cells, leading to the effective treatment of CRC [175]. In addition, they explored the potential of utilizing liposomes and other nanoparticles, such as hard nanoparticles, to provide a targeted delivery pathway for siRNAs [175]. In this way, the nanoparticles not only enhanced drug accumulation in tumor cells but also were able to encapsulate and deliver difficult-to-solubilize drugs, significantly improving therapeutic efficacy [175].

The problem of chemotherapy resistance is one of the important therapeutic dilemmas facing CRC, and oxaliplatin, as a first-line drug in chemotherapy for CRC, is an important yet to be solved challenge [176]. To address this issue, Huang *et al*. [177] developed and prepared a nanoparticle delivery system that helps to improve chemotherapy resistance and enhance the efficacy of chemotherapy. They initially found that high expression of Asporin (ASPN) was closely associated with oxaliplatin resistance as well as poor prognosis in CRC [177]. Further studies showed that down-regulation of ASPN could reverse its mediated oxaliplatin (OXA) resistance, in addition to its pro-apoptotic effects, making ASPN an important therapeutic target [177]. Taking this idea as a starting point, they constructed a nanoparticle co-delivery system (PPO-siASPN) capable of delivering both OXA and siRNA targeting ASPN (siASPN) for the chemotherapy of CRC, and verified the functional roles of the nanoparticles for reversing chemoresistance and anti-tumor in a mouse model [177]. This therapeutic strategy integrates the advantages of gene therapy, utilizing the accuracy of siRNA targeting of target genes and the effectiveness of silencing target genes to overcome the resistance to conventional chemotherapy, and provides valuable insights into the development of more effective therapeutic strategies for CRC. In addition, a study developed a novel nanocarrier system that significantly improved the efficacy of CRC treatment by combining the chemotherapeutic drug doxorubicin (DOX) and siRNA targeting CD73 to overcome cytotoxicity and resistance to chemotherapy [178]. CD73 is a molecule that plays key roles, including the promotion of tumor growth, angiogenesis, metastasis, immunosuppression, and chemotherapeutic drug resistance [179]. This study used Chitosan Lactate (CL) nanoparticles, which have surfaces functionalized by HIV-1-derived TAT peptides and Hyaluronate (HA), with the aim of increasing efficacy and reducing side effects by delivering CD73 siRNA and DOX directly to cancer cells, inhibiting CD73 while releasing DOX [178]. In this study, by co-delivering CD73 siRNA and DOX, the proliferation and metastasis of cancer cells were successfully inhibited [178]. The nanoparticles used were able to significantly reduce tumor growth and enhance the anti-tumor immune response in a mouse tumor model, which notably extended the survival rate of the tumor-bearing mice [178]. This finding provides a new strategy for gene therapy combined with nanomedicine treatment for CRC, demonstrating the great potential to overcome drug resistance and side effects through precision delivery therapy [178, 179].

Lipid nanoparticles have emerged as highly effective carriers for siRNA delivery. Gilleron *et al*. [180] developed lipid nanoparticles encapsulating traceable siRNA, enabling the visualization of siRNAs targeted transport, cellular uptake, intracellular trafficking, and endosomal escape *in vivo*. This advancement sheds light on the key challenges and mechanisms underlying siRNA delivery, marking a significant breakthrough in refining delivery strategies, augmenting the effectiveness of cancer suppression, and ultimately enhancing patient outcomes.

Lanthanum carbonate, an FDA-approved drug, is recognized for its favorable safety profile [181]. Its use does not significantly increase lanthanum levels or induce side effects in the human body post oral administration. This safety is largely due to lanthanum's capacity to bind with phosphate in the gastrointestinal tract, forming a stable lanthanum phosphate complex [181]. This binding process not only prevents the excessive absorption of phosphate but also ensures that lanthanum can be released from the complex under suitable conditions, thereby maintaining its therapeutic efficacy without compromising safety [181]. Based on the interaction law between phosphate and lanthanum, Li *et al*. [181] developed and prepared lanthanum phosphate nanoparticles (CS/LaP/siRNA NPs) loaded with siRNA encapsulated by polysaccharide chitosan, which were internalized by lattice protein-mediated endocytosis and vesicle-mediated endocytosis in an energy-dependent manner. The study [181] demonstrated that the nanoparticles (CS/LaP/siEGFR NPs) possess pH-responsive capabilities to decompose and subsequently release siRNA and lanthanide ions, facilitating gene silencing. The simultaneous delivery of these components proved effective in HT-29 CRC cells, targeting and reducing the expression of the EGFR genes. This process concurrently increased miR-34a levels, which in turn led to the suppression of downstream genes such as cyclin D1, Bcl-2, MMP-9, and MMP-1. As a result, there was a significant reduction in tumor growth and metastasis in both *in vitro* and *in vivo* models. Furthermore, these nanoparticles exhibited negligible toxic effects *in vivo*, highlighting their potential as a viable therapeutic option for CRC treatment. A study by Li *et al*. demonstrated an innovative nanomedicine delivery system that enhances targeted gene therapy and immunotherapy for cancer by efficiently delivering siRNAs targeting Stat3 using cancer cell membrane-encapsulated nanoparticles (CMDS) [182]. The study demonstrated its efficacy in delivering siRNAs targeting the Stat3 gene *in vitro* and *in vivo* and its potential to induce anticancer immune responses, highlighting the tumor-targeting properties, safety profile, and ability to elicit immune responses of cMDS, which demonstrated significant anticancer activity in tumor models [182]. The intriguing innovation lies in the study's use of cancer cell membranes to encapsulate drug-carrying nanoparticles, which exploited the natural targeting ability and immune activation of cancer cell membranes to achieve precise tumor strikes and activation of the immune system, resulting in effective inhibition of tumor growth and enhancement of the immune response [182]. Despite the innovation and effectiveness of this nanosystem in cancer inhibition strategies, issues such as its safety still deserve attention and exploration, and subsequent systematic evaluation and optimization are needed to realize its great potential in future clinical applications [182].

B-conjugated proteins have increased expression in approximately 70% to 80% of colorectal tumors and play an important role in promoting tumor progression [183]. Rudzinski *et al*. [183] used chitosan and polyethylene glycolated chitosan (PEGvlated chitosan) nanoparticles as carriers to efficiently deliver siRNAs targeting B-conjugated proteins into CRC cells. These nanoparticles have diameters between 100 and 150 nm and have an encapsulation efficiency of 97% [183]. Through Western blot analysis, they further demonstrated that siRNA contained in chitosan and polyethylene glycolized chitosan nanoparticles was able to reduce B-conjugated protein levels in CRC cells [183]. These findings suggest that the use of chitosan and polyethylene glycosylated chitosan nanoparticles as carriers to deliver siRNA is an effective strategy to successfully enter colorectal cells and reduce the levels of proteins that promote tumor progression, providing new possibilities for the treatment of CRC [183].

Liver metastasis occurs in about 50% of CRC patients, which is one of the most important causes of death in CRC patients, and the main treatment is surgical resection [184]. However, due to the hidden metastatic portion that cannot be completely resected by surgery, the long term survival rate of patients after resection of liver metastasis from CRC is about 50% [185, 186]. Therefore, early targeted prevention of CRC before it progresses to the advanced stage of liver metastasis is one of the preventive strategies to effectively circumvent the adverse outcome of death from liver metastasis and improve the prognosis. As the most abundant and only expressed miRNA in adult liver tissues, miR-122 affects gene expression in liver tissues by negatively regulating the target mRNA, CAT-1, as well as regulating other functional target mRNAs [187]. It is a key factor deeply involved in the biological functions of the liver, as well as the occurrence and development of liver diseases, and a potentially promising therapeutic target [188]. The therapeutic strategy of combining nanoparticles with miRNAs for targeted delivery of miRNAs to the therapeutic site is one of the hotspots that have been enthusiastically researched since the emergence of the nanotechnology field. The use of nanoparticles to target miR-122 with liver tissue specificity to liver tissue for the treatment of CRC liver metastasis could be an idea to maximize the advantages of both. In this vein, Sendi *et al*. [189] designed lactose-targeted lipid calcium phosphate (Gal-LCP) miRNA122 nanoparticles aimed at preventing and treating liver metastasis of CRC. Galactose in the nanoparticles was able to target the liver to mediate endocytosis in the liver allowing the nanoparticles to enter into the cells of the liver tissue. Calcium lipid carbonate protects the stability of miRNA-122 from degradation. The nanoparticles were mainly distributed in the liver tissue, which could effectively deliver miRNA-122 to the liver cells, and the miRNA-122 delivered to the liver cells was still biologically active, and a small amount of nm was distributed in the lung and kidney tissues, which did not show any obvious toxicity effect on the tissues. The experimental research [189] demonstrated that miRNA-122 delivered *via* nanoparticles could effectively inhibit the liver metastasis of CRC, thereby prolonging survival and significantly improving patient prognosis. This effect is achieved through the down-regulation of liver metastasis-related genes, enzymes, chemokines, and their receptors. Furthermore, miRNA-122 impacts the liver immune microenvironment by reducing the infiltration of immune-suppressing cells and enhancing the anti-tumor immune response.

Epigenetic modification is a mechanism that regulates gene expression without altering the DNA sequence, and RNA modification belongs to one type of epigenetic modification [190]. N6-methyl-adenosine is one of the most common genetic modifications of eukaryotic messenger RNA. One of the most common genetic modifications of RNAs characterized by its dynamic and reversible nature, plays a role in regulating various biological processes, including adipogenesis, spermatogenesis, carcinogenesis, and development [190]. As a novel epigenetic marker, it influences different aspects of mRNA metabolism, such as transcription, splicing, stability, and translation [190]. This versatility has shown great potential in biomedical research, highlighting its significance in the understanding and manipulation of cellular processes [190]. ALKBH5, an RNA m6A demethylase [191], has been found to play different roles in the context of different types of cancers: in breast cancer, glioblastoma, non-small cell lung cancer, head and neck squamous cell carcinoma and acute myeloid leukemia, it acts as an oncogene and promotes tumor progression, while in the the context of esophageal cancer, it plays a role in suppressing cancer progression, suggesting the complexity and environment-dependence of its role in cancer biology [192-195]. mRNA has emerged as an innovative gene therapy in recent years due to its unique advantages and has shown great therapeutic potential in research and clinical trials for many types of tumors [196, 197]. mRNA therapies are able to stimulate a powerful immune response to target cancer cells while avoiding the risk of genome integration that may be associated with conventional gene therapy. Despite the promising results, the use of mRNA therapies faces challenges due to the inherent characteristics of mRNA, such as its large size, high negative charge, susceptibility to enzymatic degradation, and instability [197]. These factors hinder the efficient utilization of mRNA therapies. Therefore, further research and technological innovations are necessary to overcome these obstacles, promoting the comprehensive application and maximizing the beneficial effects of mRNA therapies in tumor treatment [197]. In order to better deliver mRNA to target tissues/cells safely and efficiently, as well as to protect mRNA from degradation and to improve its intracellular delivery efficiency, a variety of emerging biomedical materials, such as nanotechnology, have been investigated and applied to various delivery systems [197]. Nanoparticles, including lipid nanoparticles, polymer nanoparticles, lipid-polymer hybrid nanoparticles, and gold nanoparticles, show a bright future in the field of mRNA delivery [197]. Future research will indeed prioritize the development of advanced mRNA delivery systems and stabilization technologies. These advancements aim to enhance mRNA stability, improve delivery efficiency, and increase cellular uptake, thereby optimizing anti-tumor therapies.

## 5 POTENTIAL APPLICATION RISKS OF NANOPARTICLES

Although nanoparticles show great potential in the diagnosis and treatment of CRC, the potential risks of its application should not be ignored. The safety and potential toxicity of nanoparticles to the human body are the first important indicators to be considered in their practical clinical application. Previous studies have shown that nanoparticles may have toxic effects on organisms, including cytotoxicity, genotoxicity, immunotoxicity, oxidative stress, apoptosis, DNA damage, and altered gene expression [198-202]. Among them, the generation of reactive oxygen species (ROS) is a common mechanism for the toxicity of various nanoparticles. ROS generated by nanoparticles interacting with organisms can induce oxidative stress, leading to DNA damage, apoptosis, and inflammatory responses occurring in cells [203]. This oxidative stress is dose-dependent and can vary based on the surface properties and functionalization of the nanoparticles. Secondly, the biocompatibility of the nanoparticles is also an important factor to be considered, whether the nanoparticles will cause an immune response or an inflammatory response. In addition, the pharmacokinetics of nanoparticles in the human body is also a central issue concerning the safety of nanoparticle applications, including its absorption, distribution, accumulation, and metabolic and excretory pathways. Accumulation of nanoparticles in the body may trigger potential toxicity, especially when they accumulate in vital organs such as the liver and kidneys [204-206]. Whether nanoparticles can be metabolized efficiently and whether the metabolites are potentially toxic and ultimately excreted safely from the body are important indicators involving the feasibility of nanoparticle applications. However, it is reassuring to know that the biocompatibility and safety of nanoparticles can be significantly improved, and their potential toxicity can be reduced or even eliminated through rational material selection, design and surface modification. Safety assessment is an important part of nanomedicine research and development, and it is expected that the safety and efficacy of nanomedicines can be improved through systematic and comprehensive evaluation as well as design improvement.

## CONCLUSION AND PERSPECTIVES

CRC remains a significant global medical challenge, with early diagnosis and treatment resistance being key factors contributing to its increased health threat. The development of nanomedicine offers a glimmer of hope in overcoming these challenges by harnessing the unique physicochemical properties of nanoparticles. These properties enable nanoparticles to exhibit superior performance and extraordinary potential in enhancing medical imaging, improving drug delivery efficiency, and achieving therapeutic targeting. Consequently, this can significantly enhance detection accuracy, improve the effectiveness of therapeutic interventions, as well as enhance patient survival rates and prognosis. Although many nanoparticles have been developed and prepared for the diagnosis and treatment of CRC, further research is required to optimize their pharmacokinetic and toxicological properties, as well as to accurately control their *in vivo* distribution and targeting. This optimization process is necessary before they can successfully pass clinical trials and gain formal approval for practical application in the clinic. Specifically, an excellent nanoparticle should possess characteristics such as low or negligible toxicity, high stability and safety, prolonged circulation time, favorable biodistribution, efficient immunoclearance, precise targeting delivery ability, and a good clearance rate, *etc*. [207]. Targeting tumor cells specifically is an ideal therapeutic strategy to maximize the efficacy of tumor-killing while minimizing damage to normal cells. Classifying tumors based on their typing and molecular expression profiles, and formulating personalized and precise therapeutic regimens for patients are key goals in improving treatment effectiveness. This approach represents the future direction for treating various types of tumors, including CRC. Therefore, it is important to discover superior molecules (ligands, antibodies, *etc*.) [208] that specifically target CRC cells and combine them with nanoparticles for precise targeting. Additionally, continuously adjusting the formulation based on *in vivo* and *in vitro* test results can help reduce toxicity and improve biocompatibility and bioavailability, ultimately achieving a balance between safety and efficacy. Balancing economic costs, optimizing preparation technology, reducing adverse environmental impacts, and improving regulatory systems are also crucial for practical application. In conclusion, nanoparticle-based strategies for tumor diagnosis and therapy hold great potential for revolutionary breakthroughs in the future.

## Figures and Tables

**Fig. (1):**
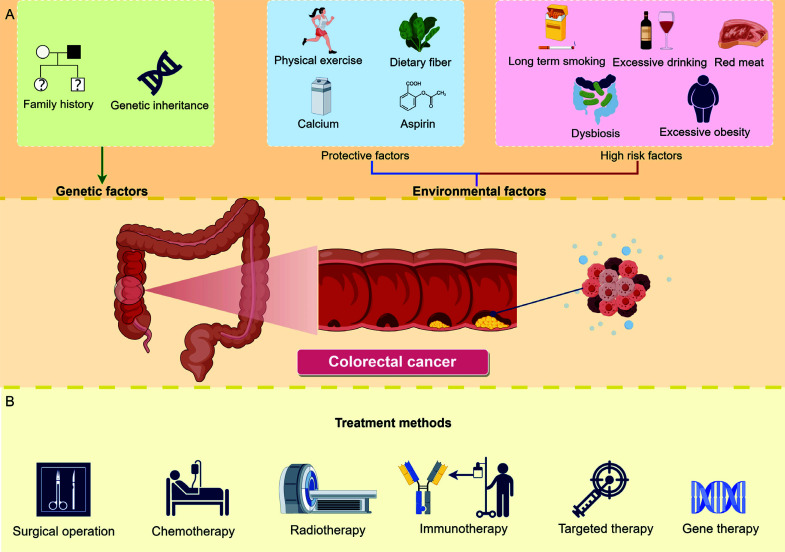
The risk factors and treatment methods of CRC. (**A**) The risk factors for CRC include environmental and genetic factors. Genetic factors involve a family history of cancer and specific genetic susceptibility genes. Environmental factors can be divided into protective factors such as exercise, fiber-rich diet, calcium supplementation, and risk factors such as smoking, excessive alcohol consumption, dysbiosis, and obesity, which suggest that we can prevent the occurrence of colorectal cancer by changing our lifestyle. (**B**) Current treatment modalities for CRC include surgery, chemotherapy, radiotherapy, immunotherapy, targeted therapy, and gene therapy.

**Fig. (2):**
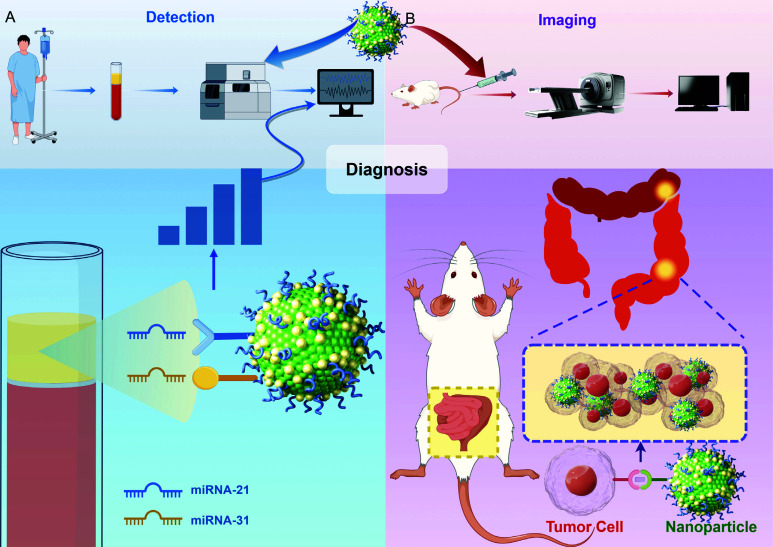
The mechanism of nanoparticle application in CRC diagnosis. (**A**) Combining nanoparticles with SERS technology can improve the effectiveness and sensitivity of detecting CRC tumor biomarkers. Collect blood from CRC patients or normal individuals and place it in a SERS biosensor for detection. At the microscopic level, the nanoparticles in the biosensor accurately target miRNA-21 and miRNA-31, which are involved in the invasion and migration of CRC, then enrich and transmit SERS signals for detection and analysis. This provides reference information for minimal invasive early screening, diagnosis, and monitoring of CRC. (**B**) Injecting tailored nanoparticles into a mouse model of CRC enables precise targeting of the cancer cells and facilitates imaging. These nanoparticles possess dual functionalities: they home in on CRC cells and enhance imaging contrast. By utilizing advanced imaging equipment, it's possible to finely visualize the location and extent of CRC within the mouse's body. This approach significantly aids in the early diagnosis and staging of the disease, offering insights that are critical for timely and effective treatment planning.

**Fig. (3):**
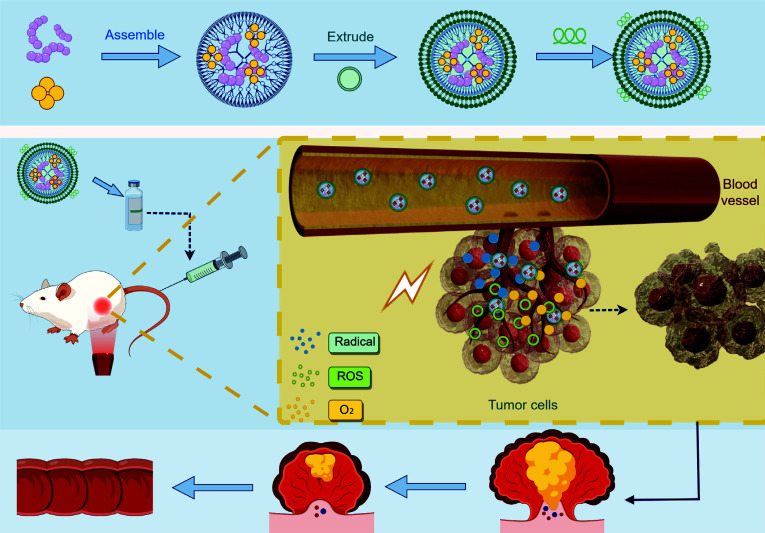
Nanoparticle synergistic PDT in CRC treatment. Specially designed nanoparticles were injected into a CRC model mouse after loading and modification. These nanoparticles are used in combination with PDT, and when irradiated at the tumor site, they are transported throughout the body through the bloodstream. When reaching the tumor site, they can efficiently target and bind to CRC cells. The activation of phototherapy promotes the production of reactive oxygen species by nanoparticles, which exerts killing power on CRC cells and effectively inhibit tumor growth. This method provides an innovative and targeted treatment strategy for the treatment of CRC. ROS: reactive oxygen species.

**Fig. (4):**
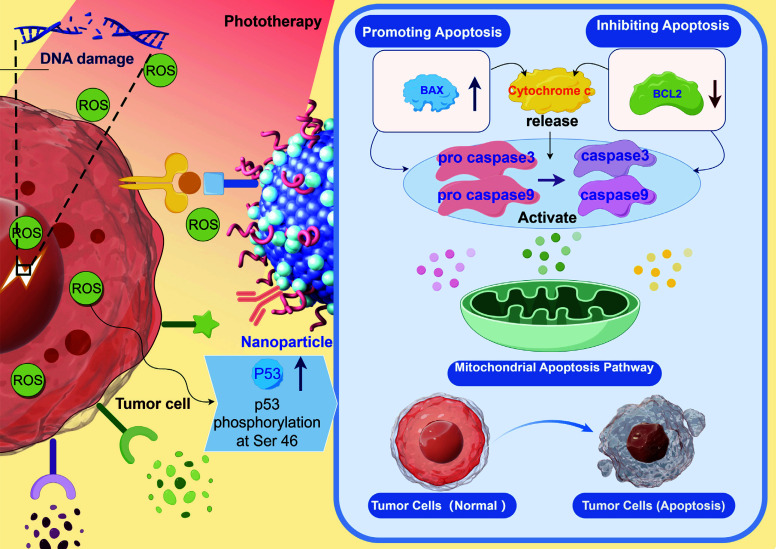
Nanoparticle Synergy with PDT Promotes Apoptosis of CRC Cells. The FA7Fe_2_O_3_ nanoparticles exploit the high-affinity interaction of folate to specifically target CRC cells, which overexpress folate receptors. Upon irradiation with the optimal wavelengths for PDT, these nanoparticles catalyze the production of substantial ROS in the tumor zone, inflicting nuclear DNA damage in the CRC cells. This damage initiates oxidative stress within the cells, impairing cellular structure and function. Concurrently, the nanoparticles facilitate the phosphorylation at the P53 ser6 site, setting off the mitochondrial-dependent apoptotic signaling cascade. This cascade involves an upsurge in pro-apoptotic protein Bax and a decline in anti-apoptotic protein Bcl2, coupled with enhanced cytochrome c release from mitochondria into the cytoplasm. These events activate caspase-9 and caspase-3, ultimately steering the cells towards apoptosis. ROS: reactive oxygen species.

## LIST OF ABBREVIATIONS

AgMNPs: Silver-coated Fe_3_O_4_ Magnetic Nanoparticles
AgNPs: Silver Nanoparticles
AP: Apoptin
ASPN: Asporin
AuNC@AuNP: Nanocage@Au Nanoparticles
AuNPs: Gold Nanoparticles
CEA: Carcinoembryonic Antigen
CL: Chitosan Lactate
CMDS: Cell Membrane-encapsulated Nanoparticles
CRC: Colorectal Cancer
CT: Computed Tomography
DOX: Doxorubicin
DSN: Duplex-specific Nuclease
EGFR: Epidermal Growth Factor Receptor
FDG: Fluorodeoxyglucose
ICD: Immunogenic Cell Death
IRIN: Irinotecan
LPS: Lipopolysaccharide
LRP-1: Lipoprotein Receptor-related Protein-1
MDR: Multidrug-resistance
MNPs: Magnetic Nanoparticles
MRI: Magnetic Resonance Imaging
mTHPC: Meta-tetrahydroxyphenylchlorin
NDKA: Nucleoside Diphosphate Kinase A
NIR-II: Second Near-infrared
OXA: Oxaliplatin
PCF-MBs: Porphyrin/Camptothecin-floxuridine Triad Microbubbles
PD-1: Programmed Cell Death-1
PD-L1: Programmed Death Ligand-1
PDT: Photodynamic Therapy
PEG: Polyethylene Glycol
PET: Positron Emission Tomography
PTT: Photothermal Therapy
RGD-TRAIL: Arginine-glycine-aspartate-tumor Necrosis Factor-related Apoptosis-inducing Ligand
ROS: Reactive Oxygen Species
SERS: Surface-enhanced Raman Scattering
VEGF: Vascular Endothelial Growth Factor
VP: Verteporfin
X-PDT: X-ray Induced Photodynamic Therapy
